# Supplementary material for: BioThings SDK: a toolkit for building high-performance data APIs in biomedical research
Source: Bioinformatics. 2022 Jan 10;38(7):2077–9. doi: 10.1093/bioinformatics/btac017 (PMC8963279; doi:10.1093/bioinformatics/btac017)

Supplemental Figures

Supplemental Figure 1: Once registered, data from a source (a data plugin) can be monitored and updated from within the dashboard


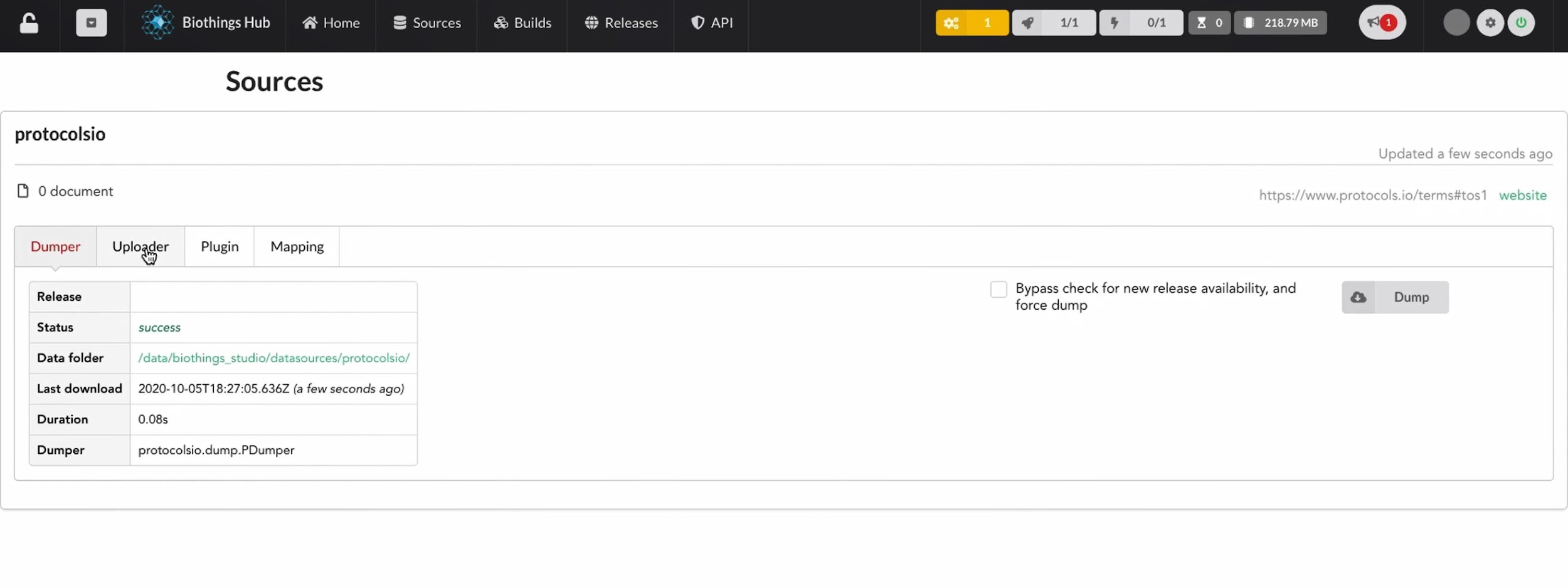


Supplemental Figure 2 - Build configurations can be created and edited directly from the dashboard


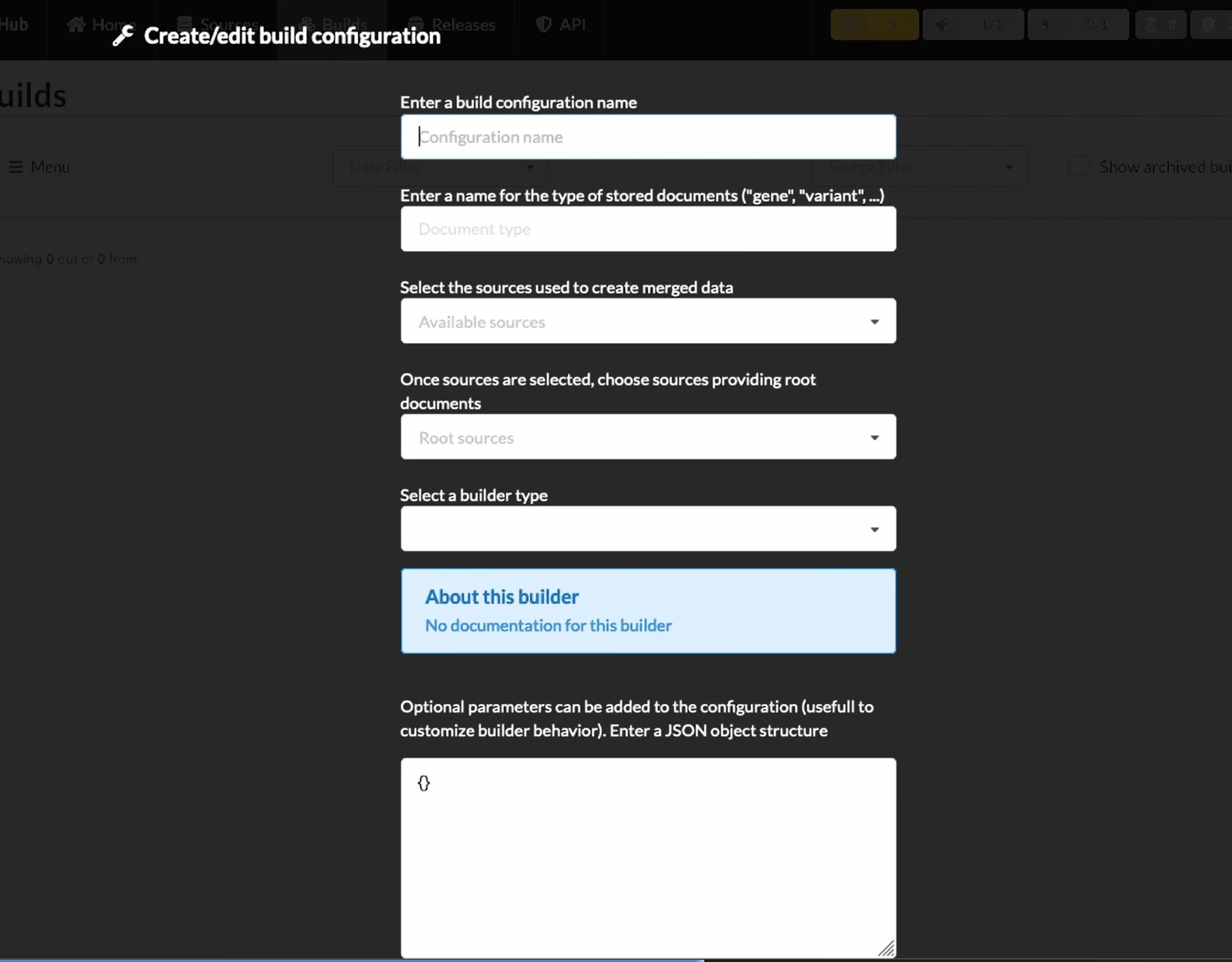


Supplemental Figure 3 - After the build is indexed by Elasticsearch, a new API can be created from within the dashboard


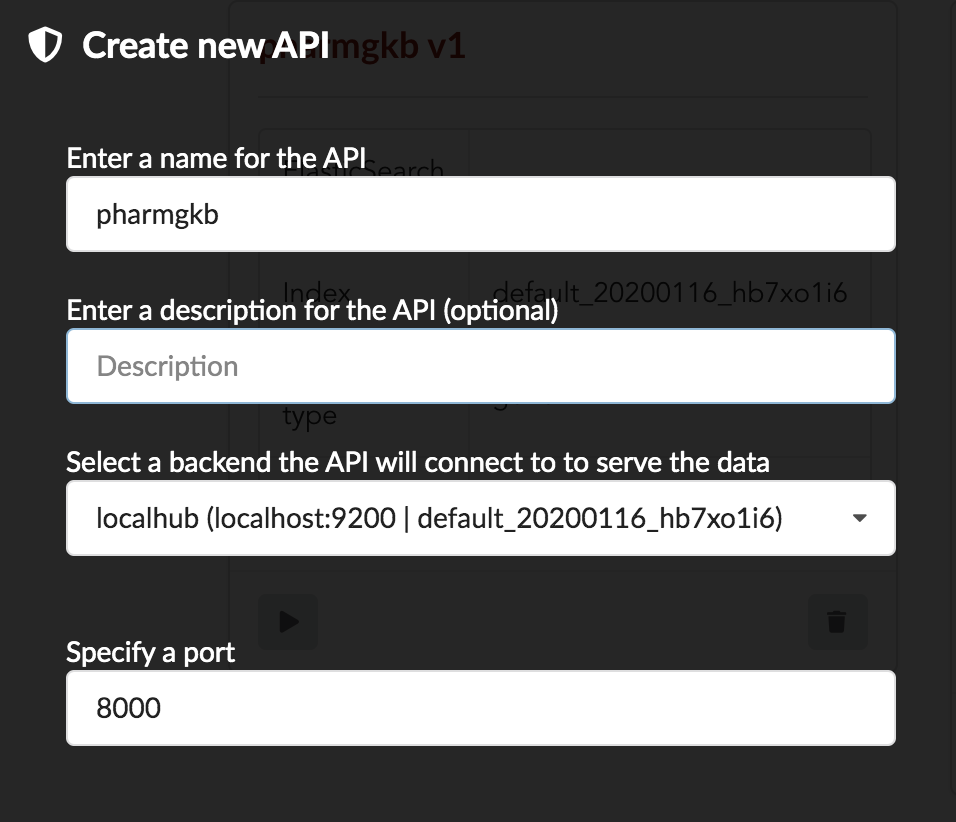

Supplement: btac017_Supplementary_Data [file btac017_supplementary_data.zip › btac017-suppl_data/Supplemental Figures.docx]
